# In Situ Forming Poloxamer-Based Thermo-Sensitive Hydrogels for Ocular Application: A Focus on the Derivatives 407 and 188

**DOI:** 10.3390/gels11090752

**Published:** 2025-09-17

**Authors:** Emanuela Longo, Elena Giuliano, Agnese Gagliardi, Valeria Gaetano, Marialaura Frisina, Mario Verdiglione, Donato Cosco

**Affiliations:** 1 Department of Health Sciences, University “Magna Græcia” of Catanzaro, Campus Universitario “S Venuta”, I-88100 Catanzaro, Italy; emanuela.longo@unicz.it (E.L.); elena.giuliano@unicz.it (E.G.); gagliardi@unicz.it (A.G.); valeria.gaetano@unicz.it (V.G.); m.frisina@unicz.it (M.F.); 2 Apotiga Laboratory, Farmacia Europea, Via Milano, 24/A, I-88100 Catanzaro, Italy; 3 ”AGreenFood” Research Center, University “Magna Græcia” of Catanzaro, Campus Universitario “S Venuta”, I-88100 Catanzaro, Italy

**Keywords:** drug delivery, in situ hydrogel, ophthalmic formulations, poloxamers

## Abstract

In ophthalmology, developing effective drug delivery systems is crucial to overcome anatomical and physiological barriers, such as rapid tear turnover and blinking, which limit the efficacy of conventional formulations like eye drops. Poloxamers, especially the derivatives 407 (P407) and 188, are amphiphilic triblock copolymers characterized by an intriguing thermo-reversible behavior, making them ideal candidates for the development of in situ hydrogels for ocular applications. Various thermo-sensitive poloxamer-based hydrogels were designed to be easily instilled as liquids at room temperature, gelling promptly upon contact with the corneal surface. These systems promoted a controlled release of active compounds, significantly improving their adhesion to the ocular surface. This review discusses the most relevant scientific literature on the topic, with particular attention to studies published in recent years. The results demonstrated that poloxamer formulations are capable of overcoming typical ocular barriers, thereby increasing drug bioavailability. The intrinsic biocompatibility of poloxamers contributes to the safety and tolerability of the system. Furthermore, P407 showed additional wound healing features. The combination of biocompatibility and thermo-reversible behavior makes poloxamer-based hydrogels a promising platform for the development of innovative ocular drug delivery systems able to enhance therapeutic efficacy and patient comfort.

## 1. Introduction

The most commonly used pharmaceutical form for ophthalmic drug administration is eye drops, typically formulated as aqueous solutions or suspensions. These are widely employed for delivering drugs intended to act on the ocular or intraocular surface. While eye drops offer certain advantages, including low cost and ease of administration, they also present significant drawbacks. These include a very short residence time on the ocular surface, poor bioavailability due to rapid elimination via the nasolacrimal pathway and, often, instability of the drug in conventional formulations [1,2]. Consequently, although eye drops are convenient, they are also among the least effective forms of ocular drug delivery. This is due to several anatomical and physiological features of the eye that limit both the efficacy and retention of the drug at the site of instillation, including tear turnover, blinking and the presence of corneal, conjunctival and blood–ocular barriers, all of which reduce drug residence time and absorption despite the availability of potent therapeutic agents [1]. The protective mechanisms of the eye, including conjunctival blood capillaries and lymphatics, nasolacrimal drainage, protein binding, systemic absorption and enzymatic degradation, further reduce drug absorption and result in only brief therapeutic effects [3]. In addition, certain physicochemical properties of the drug, such as lipophilicity, solubility, molecular weight, charge, and degree of ionization, affect both the route and the rate of corneal permeation [4]. These challenges underscore the need to develop innovative drug delivery formulations to enhance therapeutic outcomes. In this context, triblock copolymers have emerged as highly promising candidates. Their ability to self-assemble into micelles or form in situ hydrogels improves drug solubility, stability and controlled release, while enhancing retention on the ocular surface and facilitating penetration across ocular barriers [5]. Triblock polymers can be obtained by arranging hydrophilic and hydrophobic segments in different sequences, allowing the design of nanocarriers with tailored physico-chemical properties. Their chemical structure provides peculiar amphiphilic architectures that enable self-assembly [6]. In ocular delivery, such structural versatility offers various advantages: triblock copolymers protect drugs from enzymatic or oxidative degradation in the tear film, enhance retention on the ocular surface through interactions with mucin and respond to local physico-chemical conditions such as temperature, pH or ionic strength, enabling in situ gelation and controlled drug release [7]. Critical features such as gelation temperature, mechanical strength and release kinetics can be fine-tuned by adjusting the ratio of hydrophilic and hydrophobic blocks,, making them superior to other copolymers for both anterior and posterior segment therapies [8,9]. While this review will specifically focus on poloxamer-based triblock copolymers due to their extensive application in ophthalmology, it is important to note that other systems, such as PLGA-PEI-PEG (poly-lactic-co-glycolic acid- poly-ethyleneimine- polyethylene glycol) have also shown promising results in ocular applications, including antifungal therapy as was true in the case of natamycin-loaded thermosensitive nanoparticle–hydrogel composites proposed for the treatment of *Fusarium solani* [10].

## 2. Approaches to Ophthalmic Treatment

### 2.1. Physiological Limitations Affecting Therapy

**Figure 1:**
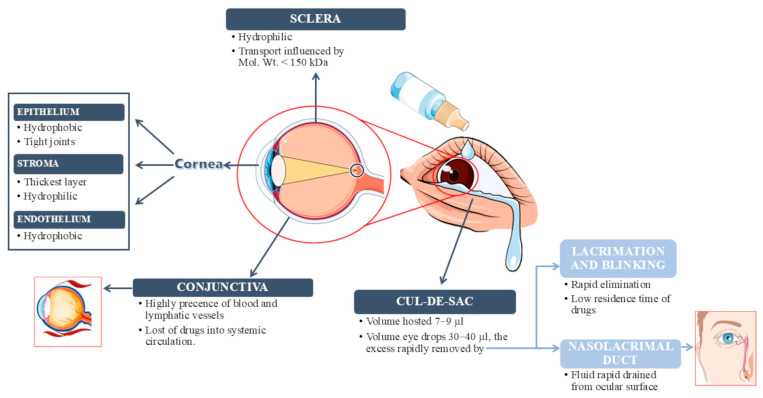
Schematic representation of the main ocular barriers to the topical application of drugs. The figure was created using images from Servier Medical Art (https://smart.servier.com/ accessed on 10 June 2025).

Ocular therapy presents a significant challenge for doctors and pharmacologists due to the various anatomical barriers within the eye. Structurally, the eye is divided into two main parts: the anterior segment and the posterior segment, each containing multiple layers that act as biobarriers, limiting drug penetration. The anterior segment accounts for about one-third of the eye and consists of the cornea, aqueous humor, iris, lens and ciliary body. The pupil is also part of this region and it acts as a window into the integrity of a complex network of ocular structures through its multifaceted functions [11]. The posterior segment, which accounts for the remaining two-thirds, consists of the vitreous humor, retina, choroid, macula, optic nerve and sclera, forming the bulk of the ocular anatomy [12]. Structures like the conjunctiva, cornea, sclera and retina, along with the blood–retina barrier, serve as static barriers to drug delivery, while dynamic barriers include factors such as tear dilution, blinking, nasolacrimal drainage, blood flow and lymphatic clearance [5]. Various parameters and challenges must be taken into account depending on the route of administration of active compounds. This review will focus specifically on the topical application of drugs (Figure 1).

**Figure 2:**
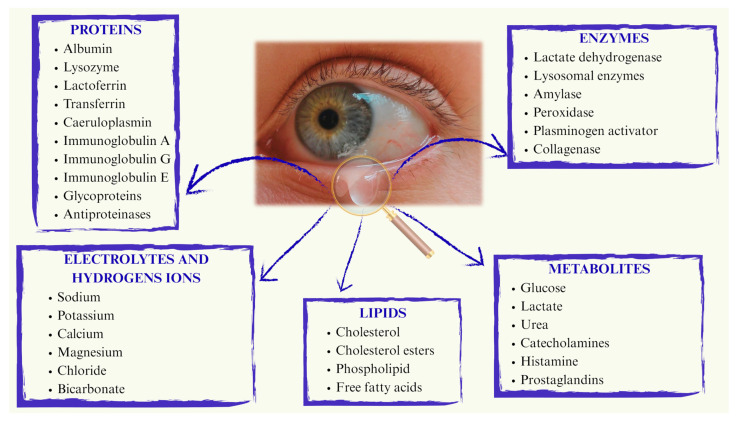
Schematic representation of tear composition.

Following topical instillation, drug absorption occurs through either the corneal or non-corneal route, depending on the molecule’s chemical properties. The cornea, composed of the epithelium, stroma and endothelium, plays a critical role in protecting the eye under physiological conditions by acting as a barrier against pathogens and exogenous agents [13]. Each layer acts as a barrier to molecules with different origins and chemical properties, with the epithelium and stroma posing greater obstacles than the endothelium [14]. The epithelium is largely impermeable to topically administered hydrophilic drugs due to its lipophilic nature. Additionally, the tight junctions between corneal epithelial cells act as a selective barrier, allowing the permeation of small molecules while preventing the diffusion of macromolecules through the paracellular pathway [15]. In contrast, the stroma, which is highly hydrated due to its lamellar arrangement of fibrils and collagen, is permeable to hydrophilic drugs and it hinders the diffusion of lipophilic molecules reinforcing the lipophilic–hydrophilic dual barrier nature of cornea [1]. The conjunctiva is a vascularized mucous membrane that lines the inner surface of the eyelids and extends over the anterior part of the sclera up to the cornea. Both the conjunctival and corneal surfaces are coated with mucin, which helps to moisturize, cleanse and protect the eye from pathogens. Ocular bioavailability is further reduced by drug loss into the systemic circulation, facilitated by the conjunctival blood and lymphatic vessels [16,17]. With respect to the sclera, trans-scleral administration is more straightforward due to its high hydration and permeability to water-soluble molecules. In fact, the sclera is generally more permeable than both the cornea and conjunctiva [18]. However, molecules with a molecular weight of 150 kDa or higher face greater challenges in crossing the sclera and exhibit slower clearance compared to low molecular weight compounds [19]. Additionally, systemic drug administration to ocular tissues can occur via intravenous or oral routes. However, oral administration is limited by drug passage through the gastrointestinal tract. The retina, meanwhile, acts as a physical barrier that affects the distribution of active compounds in both the anterior ocular tissue and the posterior segment of the eye, due to the presence of the inner blood–retinal barrier (BRB) and the retinal pigment barrier (RPB) [20]. In the context of ocular physiology, ocular surface temperature and tear film sensitivity are crucial and closely interconnected parameters. In healthy eyes, ocular surface temperature values, measured immediately after blinking are typically 34–35 °C, and then progressively decrease in the interblink interval. Cyclosporine has been shown to display retrograde solubility in aqueous media and solid-state transformations that are accelerated at higher temperatures, while prostaglandin analogues such as bimatoprost, latanoprost and travoprost demonstrate notable thermal instability under conditions simulating daily use [21,22]. Taken together, these findings suggest that the ocular surface temperature range may critically influence both solubility and stability of topically applied drugs, underscoring the need to consider local thermal conditions in ophthalmic formulation design. Ocular temperature is influenced by a variety of factors, including environmental and body temperature, inflammation and blinking itself, but it is also significantly related to tear film parameters [23]. After topical administration, the drug initially mixes with the tear film, which consists of ions, lipids and proteins (Figure 2).

Despite the development of effective therapies, the drug’s contact time with ocular tissues remains a significant challenge. The continuous production of tear fluid and its rapid drainage through the upper and lower canaliculi drastically limit how long a drug can exert its function on the ocular surface [24]. For this reason the tear film viscosity plays a key role in the mean residence time of a drug in the eye. Its composition directly influences the stability and permanence of active compounds in the administration site. Although it was previously believed that mucins were solely responsible for this viscosity due to their ability to form a hydrogel network, more recent studies revealed that viscosity is related not only to a single component but rather to complex molecular interactions between secretory and membrane-associated mucins, lipids and several proteins. This multifaceted and intricate nature of the tear film represents a criticism to be evaluated when novel formulations are developed; in fact, altering viscosity with the aim of prolonging the drug contact time without compromising ocular function is a challenge [25].

Lacrimation and blinking are effective protective mechanisms that help keep the eye clear of external substances but also hinder efficient ocular drug delivery [3]. Additionally, the volume of the conjunctival cul-de-sac, which typically holds around 7 μL of tear fluid, is much smaller than the volume of a standard eye drop (30–40 μL). As a result, the blink reflex immediately expels the excess fluid that the eye cannot retain, leading to a rapid decrease in drug concentration within just a few minutes [26,27]. Moreover, the tear turnover is approximately 1 μL/min, which contributes to ongoing drug loss from the ocular surface [28]. As a result, a significant portion of topically applied drugs is immediately diluted in the tear film, with excess fluid spilling over the lid margin, while the remainder is rapidly drained into the nasolacrimal duct. Notably, it is estimated that less than 5% of the administered dose reaches the target tissue via topical application [1].

### 2.2. Ocular Drug Delivery Systems

Ocular drug formulations are categorized into liquids (solutions, suspensions, and emulsions), semi-liquids (ointments and gels) and solids (ocular inserts, contact lenses, etc.) [29,30]. The primary route of ocular drug administration is topical; however, periocular and intraocular administration, involving the use of injections and implants, is also employed [29]. The topical route is generally preferred due to its non-invasive nature, the relatively low incidence of side effects and improved patient compliance. Topical ophthalmic solutions are utilized in the treatment of eye injuries, but these formulations typically require multiple daily applications to maintain a therapeutic drug concentration at the site of administration [31]. Various ocular diseases can affect both the anterior and posterior segments of the eye. While anterior-segment conditions are more common and directly exposed to the external environment, posterior-segment diseases, such as age-related macular degeneration (AMD) and diabetic retinopathy, pose a greater treatment challenge when using topical therapies [32]. The high sensitivity of eye, along with its biological barriers, has attracted significant research interest, as these factors can compromise the therapeutic effectiveness of drugs [16].

Given the aforementioned challenges, a sufficient therapeutic concentration can only be achieved through frequent instillations of conventional eye drops. However, this approach may increase the risk of side effects due to the absorption of the drug through blood vessels in the conjunctival stroma or the nasal mucosa [33]. To address these limitations, various ophthalmic drug delivery systems, such as hydrogels, nanoparticles, liposomes, in situ gels and micelles have been developed to optimize the localization of active compounds at the target tissue in the eye. All conventional pharmaceutical formulations have inherent limitations in ophthalmic therapy [31]. For these reasons, in the last decade, scientific research has focused on evaluating technologies that encapsulate drugs within biocompatible systems. These systems aim to increase drug residence time, minimize clearance mechanisms in the eye and reduce the side effects associated with conventional eye drops [34]. The design of new pharmaceutical forms must, of course, adhere to the standard characteristics of conventional eye drops, such as isotonicity, appropriate pH, adequate viscosity and surface tension. Additionally, attention must be given to bioadhesion, particularly to polymers such as cellulose derivatives, polyvinyl alcohol, polyacrylic acid, chitosan and hyaluronic acid, which can effectively interact with mucin [35].

The advantages of ocular drug delivery systems can be summarized as follows:

1. Patients can self-administer the treatment, leading to improved compliance;
2. Enhanced stability of the drug encapsulated within the formulation;
3. Reduced drug elimination due to increased residence time on the ocular or intraocular surface or in the conjunctival sac, as well as improved absorption through the corneal cells;
4. The ability to modulate drug release, reducing the frequency of instillations and minimizing drug loss from the precorneal area;
5. The potential for polymer manipulation to achieve specific tissue targeting [36].

It is crucial to emphasize that the topical application of drugs is not limited to treating conditions affecting the anterior structures of the eye, such as inflammation and infections [9]. In fact, hydrogels, made of different materials, have also been developed for the treatment of glaucoma [37,38], a pathology characterized by high intraocular pressure (IOP) that induces mechanical stress on various structures of the anterior and posterior chambers [39]. An example is the nifedipine-containing hydrogel, made up of P407, P188 and hydroxypropyl methylcellulose K4M, which in a preclinical study demonstrated a significant reduction in IOP when compared to commercially available conventional eye drops [40].

In consideration of the aforementioned aspects, this review is focused on the most recent developments in drug delivery systems intended for topical ophthalmic therapy, with particular attention paid to hydrogels formulated from polymers, such as poloxamers, characterized by thermo-responsive properties.

Relevant keywords such as in situ hydrogels, ocular application, poloxamer 407, poloxamer 188 and their association, etc., have been used in order to collect the research and review articles mainly published between 2020 to 2025 and available on Google Scholar, PubMed and Scopus.

Hydrogels employed in ocular drug delivery applications have exhibited promising features, facilitating the sustained and controlled release of therapeutic agents. Moreover, their high water content closely resembles the natural ocular environment, thereby ensuring biocompatibility and underscoring their potential utility in the treatment of ocular diseases [41].

## 3. Hydrogels

### 3.1. General Properties of Hydrogels

In 1960, Wichterle and Lim pioneered the development of a synthetic hydrogel composed of poly-2-hydroxyethyl methacrylate (PHEMA), which was later adopted as a filler following eye enucleation and in the fabrication of contact lenses [42]. Hydrogels constitute three-dimensional polymeric matrices comprising natural or synthetic polymers (Table 1), which assemble via physical or chemical cross-linking and are characterized by diverse properties including flexibility, biodegradability and biocompatibility [26]. Within their polymeric network, hydrogels exhibit a variety of functional groups, such as -NH_2_, -COOH and -OH, which facilitate the absorption of substantial quantities of water and regulate the swelling behaviour of the system. Notwithstanding their significant hydrophilicity, hydrogels undergo swelling but retain their structural integrity in the medium due to the presence of cross-links, encompassing covalent, hydrogen, Van der Waals interactions and ionic interactions, the latter being particularly relevant for polyelectrolyte hydrogels [43].

These chemical interactions, augmented by physical entanglement, permit the entrapment of a range of active compounds [44,45].

The encapsulation of drugs within hydrogels can be achieved through two principal methods:

- Post-loading: this involves the entrapment of the drug molecule within the established three-dimensional network of the hydrogel matrix;
- In situ loading: in this approach, the drug forms interactions with the solution or the polymer before the hydrogel structure is created [46].

The development of hydrogels can utilize both natural and synthetic polymers. Nevertheless, the use of natural polymers can present complexities owing to their inherent limitations in stability and mechanical resistance. Indeed, synthetic polymers typically possess greater mechanical and chemical strength than natural polymers [47]. Such resistance, attributed to the robust bonds within their matrix, prevents the premature degradation of the systems. As a result, the utilization of synthetic polymers is often favored due to their enhanced susceptibility to chemical and physical manipulation [44].

Biodegradable and hydrophobic synthetic polymers, characterized by low toxicity and minimal side effects, represent the most commonly employed materials in the development of smart hydrogels for drug delivery applications. This facilitates the localized, sustained and prolonged release of the therapeutic agent, thereby diminishing the requisite number of administrations [45,46]. This review will focus on the development of synthetic hydrogels, obtained through the polymerization of monomers such as PEO and PPO, for ocular applications.

Interest in hydrogels has increased significantly in the last decade due to their potential smart properties [48]. Indeed, depending on their polymer composition, hydrogels can exhibit a response as a function of a specific stimulus. These stimuli-responsive properties can enable the development of formulations useful for drug targeting and the controlled release of bioactives [49]. Polymer-based gels are categorized into two groups: bioadhesive gels and in situ forming systems. These formulations serve to enhance bioavailability and mitigate side effects associated with the systemic absorption of topically administered ophthalmic drugs. Bioadhesive gels present as viscous solutions prior to ocular application and are commonly employed in the treatment of dry eye syndrome as a substitute for natural tears. These ophthalmic solutions do not undergo any alterations following their administration [50,51].

Bioadhesive ophthalmic solutions commonly exhibit several limitations, including a lack of consistent accuracy and reproducibility in administration. Moreover, they may cause patient discomfort, transient blurred vision and reflex tearing, which can further compromise the vision. As a result, their application is frequently restricted to the treatment of dry eye disease, serving as artificial tears [52]. The polymers incorporated into these formulations are predominantly cellulose derivatives, such as hydroxypropyl methylcellulose (HPMC), which serve to enhance viscosity, mucoadhesion and the stability of the formulation. Furthermore, polymers such as hyaluronic acid (HA) are employed to exploit their pseudoplastic characteristics, thereby ensuring effective diffusion of the formulation across the ocular surface [53]. In situ gelling systems are liquid formulations applied to the eye in drop form and subsequently undergo a sol-to-gel phase transition within the conjunctival cul-de-sac [54].

### 3.2. In Situ Hydrogels

Contemporary research has increasingly concentrated on in situ hydrogel formation as a means of mitigating the limitations inherent in conventional ophthalmic solutions. In situ hydrogels are developed as polymeric solutions capable of being administered in liquid form and subsequently undergoing a phase transition to a semisolid gel upon exposure to physiological environments. The in situ gelation system constitutes a particularly interesting strategy and a promising approach for prolonging the residence time of drugs on the ocular surface and improving their bioavailability [55]. In situ forming hydrogels are capable of increasing viscosity in response to alterations in pH or temperature within the precorneal region, thereby resulting in decreased naso-lacrimal drainage, extended ocular absorption time and enhanced bioavailability. A notable advantage of this formulation type is its liquid state at ambient temperature, permitting simple instillation as an ophthalmic drop, with subsequent gelation occurring as a function of a specific physiological parameter. This behavior allows the formulation to resist shear forces within the conjunctival cul-de-sac and to impede excessive dilution by lacrimal fluid [56]. The preparation of in situ hydrogels involves the dissolution of a suitable polymer in an appropriate solvent. The selection of the solvent is contingent not only upon the solubility of the polymer but also upon the biocompatibility and overall stability of the delivery system. Moreover, these systems are characterized by a facile preparation procedure, typically consisting of a single step of mixing and solubilization of the constituent components and cost-effective. This ensures the uniformity and reproducibility of the resultant product [57]. The polymers employed in the development of in situ hydrogels exhibit reversible phase transition characteristics and pseudoplastic behaviour, a property useful for minimizing interference with the natural act of eye blinking. The extended ocular residence time of these systems promotes a sustained release of the active compounds, resulting in enhanced bioavailability and a decrease in the number of administrations required, thereby leading to improved patient compliance and comfort, without inducing blurred vision [54]. The instillation of an in situ hydrogel can favour a decrease of the drug concentration used and administration times, consequently reducing the potential side effects and toxicity of several active compounds [58]. However, hydrogels present certain drawbacks that can hinder their use; among them, for example for hydrophobic drugs, the low loading capacity and distribution in the polymeric matrix of the molecules can compromise the administration of various active compounds. Furthermore, stability criticisms and the microbial contamination are other problems to be properly investigated [59].

### 3.3. Thermo-Sensitive In Situ Hydrogels

Within the spectrum of pharmaceutical formulations, thermo-sensitive hydrogels have been shown to be systems capable of promoting a sustained release of the active compounds over an extended period [60]. Temperature-sensitive polymers, characterized by the presence of both hydrophilic and hydrophobic domains within their structure, exhibit a phase transition that is dependent on temperature fluctuations and modulates their solubility.

In particular, changes in temperature affect the interplay between the hydrophilic and hydrophobic residues of the polymer and water molecules, thereby altering the solubility of the cross-linked network and inducing the sol–gel phase transition. Temperature-sensitive hydrogels can demonstrate either a positive response, wherein the gel material swells upon an increase in temperature, or a negative behavior, wherein the material undergoes shrinkage as the temperature rises [61]. Thermo-sensitive hydrogels demonstrate volume phase transitions or sol–gel phase transitions at critical temperatures, specifically the lower critical solution temperature (LCST) or the upper critical solution temperature (UCST). Polymers exhibiting LCST undergo a transition from swelling to shrinking (or sol to gel) upon an increase in temperature, whereas UCST systems undergo the inverse transition [62]. Polymers that exhibit LCST phase separation phenomena are more prevalent and advantageous compared to polymers characterized by UCST properties. Thermo-responsive systems at temperatures below the lower critical solution temperature (LCST) exist as transparent, homogeneous, free-flowing polymer solutions and undergo a transition to a cloudy state upon reaching the LCST [63]. The necessity of high temperatures for mixing UCST polymers and drugs can, in fact, prove detrimental to the activity of thermo-sensitive compounds. Conversely, hydrogels exhibiting LCST behaviour, particularly those with a sol–gel transition temperature of approximately 37 °C, have been extensively investigated as carriers for cells, drugs and biomolecules, as they permit their incorporation under mild conditions. A representative example of such biocompatible thermo-reversible LCST hydrogels is composed of poloxamers [64].

The application of these considerations to ophthalmic formulations leads to varying outcomes. The limited therapeutic response of conventional ophthalmic solutions, often due to precorneal drug elimination, can be overcome by administering in situ hydrogels. These hydrogels are prepared from polymers that exhibit reversible phase transitions and pseudoplastic behaviour to minimize interference with blinking [29]. Subject to specific conditions, the formulation undergoes an in situ phase transition, resulting in the formation of a gel that exhibits resistance to shear forces within the conjunctival cul-de-sac, thereby promoting drug release under physiological conditions. In situ gelation systems demonstrate an increase in viscosity as a consequence of the temperature within the precorneal region [56]. Subsequent to the instillation of the aqueous solution containing polymers responsive to external stimuli, a viscous and mucoadhesive hydrogel is formed on the surface of the eye, leading to an increase in ocular retention time and the bioavailability of the incorporated drug [35].

**Figure 3:**
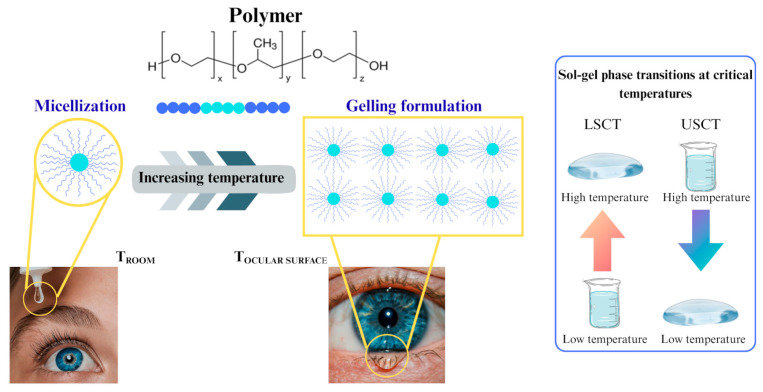
Schematic representation of the in situ gelation mechanism of a P407 aqueous solution in the eye. Red arrow: increasing temperature; blue arrow: decreasing temperature.

Thermo-responsive polymer-based systems exhibit a liquid state at ambient temperature (20–25 °C) and undergo gelation at approximately 35 °C, which corresponds to the temperature of the ocular surface (Figure 3). This characteristic facilitates their easy instillation as ophthalmic drops without inducing irritation or blurred vision, with subsequent gelation occurring upon interaction with the eye [65]. In accordance with these principles, a thermosensitive hydrogel composed of 20 wt% P407 was formulated for the delivery of progranulin (PGRN). This macromolecule is a protein exhibiting anti-inflammatory, regenerative and re-epithelializing characteristics relevant to corneal tissue. Yan et al. [66] demonstrated that P407 is a thermo-responsive polymer of the LCST type in solution, forming micelles at low material concentrations and hydrogels at elevated temperatures (≥LCST). At a temperature of 4 °C, the hydrogel exists in the form of a solution, thereby allowing for its admixture with the protein. This physical state was preserved at ambient temperature and the formulation was instilled into the eye as ophthalmic drops, undergoing a transition to a hydrogel at 34.5 °C. This physical transition of the system favoured the slow and sustained release of PGRN, while the formulation also functioned as a protective barrier for the cornea.

## 4. Poloxamers

### 4.1. General Properties of Poloxamers

Poloxamers, by virtue of their thermosensitive properties, have been the focus of research for numerous years, with a particular emphasis on drug delivery applications. Furthermore, they are also employed as non-ionic surfactants and cross-linking agents [27]. Poloxamers, trade name Pluronic^®^, are triblock polymeric structures composed of two hydrophilic blocks, such as PEO, and a central hydrophobic block, such as PPO. Owing to this amphiphilic nature, poloxamers exhibit surfactant properties and the capacity to interact with hydrophobic surfaces and biological membranes. In aqueous solutions, the amphiphilic character of these copolymers leads to self-aggregation of the macromolecules into micelles upon reaching the CMC (critical micellar concentration). PPO portions aggregate to form hydrophobic micellar cores surrounded by hydrophilic PEO coronas able to interact with water-soluble molecules. This mechanism leads to thermo-responsive gelation and the formation of a three-dimensional micellar network [67]. The obtained system absorbs water and swells upon contact with tear fluid, inducing a variation of the pore size and diffusional pathways, thereby modulating the release of the entrapped drugs. The relative balance between hydrophilic and hydrophobic domains determines gel density, erosion behaviour and release kinetics. This interplay of micellization, swelling and erosion provides a robust rate-controlling mechanism that sustains therapeutic levels compared with conventional eye drops [68,69]. The thermo-sensitivity of poloxamers is determined by their solubility in water. While PEO is water-soluble even at low temperatures, PPO is not. Consequently, the PEO/PPO chain ratio dictates the thermo-sensitivity. The distinctive properties of poloxamers have been discussed in numerous review articles and will not be detailed in this manuscript [60,70]. Polymer concentration represents an additional parameter capable of influencing the phase transition of poloxamer-based systems. Indeed, poloxamers at physiological temperature typically undergo gelation at concentrations greater than 15%. Conversely, formulations characterized by an excessive polymer content can elicit undesirable effects. For example, intraperitoneal administration of P407 has been shown to induce significant hypertriglyceridemia and hypercholesterolemia; this effect is dose-dependent and does not occur at lower doses [71].

Gugleva et al. [72] developed an in situ poloxamer hydrogel with the capacity to retain niosomal doxycycline. They exploited the interaction between the PEO/PPO residues and the corneal surface to augment the residence time of the bioactive agent. Additionally, it was demonstrated that the mucoadhesion and phase transition temperature could be modulated through the incorporation of HPMC. The optimal formulation, characterized by a favourable phase transition temperature of 34 °C, was determined to be composed of 15% P407 and 1.5% HPMC. The temperature also exerts an influence on this process; specifically, upon reaching the critical micelle temperature (CMT), the dehydration of the PPO blocks commences, leading to the formation of multichain spherical micelles [72].

Natural antioxidant agents, such as resveratrol, exert a scavenging activity against free radicals and various derivatives are characterized by antibacterial properties [73,74]. Nevertheless, these molecules may exhibit poor aqueous solubility often leads to low permeation and rapid degradation. To address these limitations, poloxamer 407-based micelles and poloxamer 407/β-casein nanoaggregates have been developed [75]. Mixed micelles may possess a low CMC and promote significant stability against both physical and chemical agents. Both types of micelles demonstrated an excellent capacity for resveratrol retention, enhancing its apparent solubility (by more than 50 times) and preserving its antioxidant capacity [76]. However, mixed micelles exhibited slightly increased toxicity, and therefore, P407 micelles were proposed as useful systems for the ocular application of resveratrol [75].

Sangitra et Pujala [77] demonstrated that poloxamers, when exceeding their CMC and at temperatures above their CMT, are capable of forming a denser gel network, characterized by increased resistance to deformation as a consequence of aggregation phenomena.

In a separate experimental investigation, artemisinin-loaded micelles with a size of approximately 50 nm, composed of 8% polyvinylpyrrolidone (PVP K90) and 4% P407, were developed. These micelles enhanced both the solubility and corneal permeation of artemisinin and have been proposed as a therapeutic strategy for age-related macular degeneration (AMD) [78].

Padaga et al. [79] described an innovative system composed of a novel synthesized copolymer. Specifically, the FCOL derivative was obtained by carboxylation of P407 with succinic anhydride, followed by the reaction with the amino groups of chitosan oligosaccharide lactate (COL). Chitosan was selected for its antibacterial activity and mucoadhesive nature, attributed to the positive charges of the polysaccharide that can interact with the negative charges on the corneal surface. This new copolymer was used to create a gel containing gatifloxacin.

Furthermore, mixed micelles can be formed utilizing two distinct poloxamers, as exemplified by systems proposed for the entrapment of fexofenadine, a drug with limited aqueous solubility employed in the treatment of allergic conjunctivitis. The drug was encapsulated within nanoaggregates composed of Pluronic^®^ 127 and 123, and enriched with Phospholipon 90G with the aim of augmenting their thermodynamic stability as a consequence of strong hydrophobic interactions with the PPO blocks. The optimized formulation demonstrated a high entrapment efficiency and a sustained release profile of the active compound retained within the inner core of the micelles [80].

Polymeric micelles can also be utilized to address certain critical issues associated with contact lenses, including water content, wettability and optical properties. Experimental investigation revealed that micelles composed of P407 and Tween^®^ 80, incorporating cyclosporine, were capable of increasing the water content, optical transmission and wettability of contact lenses [81]. Furthermore, they promoted a sustained release of cyclosporine, lasting approximately 120 h and enhanced its ocular absorption [81].

Poloxamers 188 and 407 represent the most widely utilized members of the United States (U.S.) FDA-approved Pluronics^®^ material classes for human use [61].

### 4.2. Poloxamer 407 Hydrogels for the Treatment of Ocular Diseases

Poloxamer 407 (P407), commercially known as Pluronic^®^ F-127, comprises 70% PEO blocks, which contribute to its hydrophilic characteristics. P407 is a member of a family encompassing more than 30 copolymers characterized by an ABA block structure, wherein a hydrophobic PPO block is positioned between two hydrophilic PEO residues. This non-toxic copolymer possesses a molecular weight of 12,000 Da, exhibits low viscosity and demonstrates the ability to form gels at physiological temperatures [82]. P407 demonstrates superior solubility in cold water compared to hot water, a phenomenon attributed to the increased formation of hydrogen bonds at lower temperatures and exhibits high stability [83]. Despite the potential benefits, poloxamers face significant challenges that hinder their widespread use. These include non-ideal mechanical properties, reduced viscosity and gel strength, accelerated erosion and insufficient bio-adhesion that require the association with other polymers such as chitosan, HPMC, carbopol, or other poloxamers such as P188 [84,85].

Li et al. [86] developed a thermo-responsive nanosuspension hydrogel for the treatment of uveitis. Carboxymethyl chitosan was employed to stabilize clobetasol propionate obtaining a nanosuspension that was integrated into a hydrogel made up of P407 and sodium alginate [86].

P407 forms an optically clear and transparent gel at the physiological temperature of the ocular cavity and its self-assembling characteristics facilitate the administration of both hydrophilic and lipophilic drugs, favoring a controlled release profile over time. Nevertheless, P407 exhibits limited structural stability at low temperatures and concentrations where micellar structures are unstable. Consequently, it is typically combined with other excipient [87].

Hydrogels composed of P407 have been proposed as drug carriers for the treatment of various diseases, including dry eye, a pathology associated with inflammation and induced by oxidative stress [3,88].

**Figure 4:**
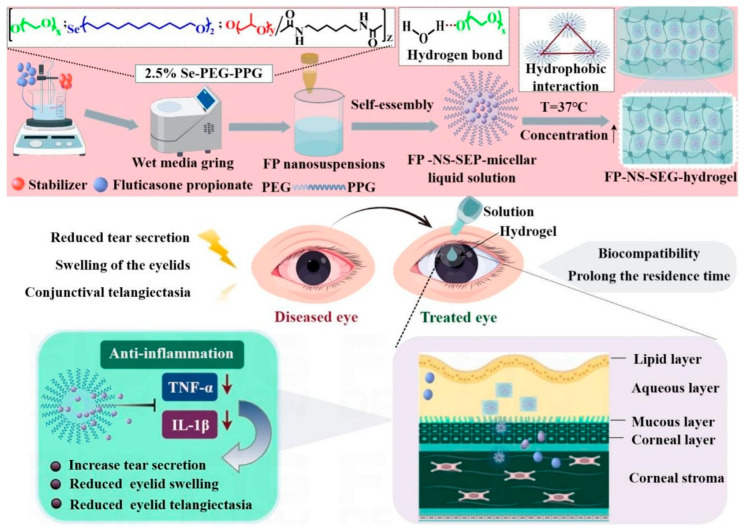
Representation of the preparation of carboxymethyl chitosan-modified fluticasone propionate nanosuspensions loaded into a thermo-responsive in situ gel for blepharitis treatment. Reproduced with permission from [89]. Copyright (2025) Elsevier.

Inflammatory eyelid conditions could also benefit from the treatment with in situ hydrogels due to the opportunity to overcome the limited retention of conventional eye drops provided by these formulations. In this regard, a fluticasone propionate nanosuspension modified with carboxymethyl chitosan was developed and encapsulated within a synthesized P407-based gel, named SEP (Figure 4), synthesized from selenol, PEO and PPO. Considering that selenium is a vital trace element that exerts a protective influence on the cells of ocular surface, it was hypothesized that the use of selenium could alter the intermolecular forces, allowing for gelation even at low concentrations. The proposed formulation offers several advantages, such as a more practical and effective therapeutic option for blepharitis [89].

A novel formulation combining uniform, ultrasmall (approximately 3 nm in diameter) antioxidant copper-selenide (Cu_2-x_Se) nanoparticles and aldehyde-modified Pluronic^®^, (AF127) hydrogels was used as eye drops and tested in an in vivo mouse model of dry eye disease (DED) [90]. These nanoparticles exhibited the capacity to neutralize ROS, thereby exerting antioxidant and antiapoptotic effects through a mechanism that mimics the activity of superoxide dismutase and glutathione peroxidase. Additionally, aldehyde residues were incorporated into F127 to facilitate the formation of Schiff bases with mucin constituents on the ocular surface, which is intended to enhance the bioavailability of the nanosystems. The formulation comprising 20% AF127 with incorporated Cu_2-x_Se nanoparticles presented a gel-forming temperature of 36.8 °C [90].

Tacrolimus is another drug used to treat dry eye syndrome. However, it is an immunosuppressive compound that can also be employed for the treatment of other significant conditions, such as corneal allograft rejection, immunogenic inflammatory diseases of the ocular surface and allergic conjunctivitis [91,92]. To enhance its delivery, an in situ gelling formulation composed of P407 and chitosan was developed, demonstrating increased drug retention on the ocular surface [93].

Nonetheless, P407 has limited mucosal adhesion and, when used at high concentrations, may induce hypertriglyceridemia in the eye. To mitigate these drawbacks, it is combined with HPMC, a polymer commonly used to improve eye hydration (particularly in glaucomatous dry eye patients), increase viscosity and enhance the comfort of eye drops [33,94].

A similar formulation, consisting of 10% *w*/*v* P407 and 0.725% *w*/*v* HPMC, incorporating chloramphenicol, was also developed [95]. More recently, however, chloramphenicol was encapsulated within chitosan nanoparticles embedded in a thermo-sensitive in situ gel system composed of 20.5% *w*/*v* P407 and 5% *w*/*v* P188. The resulting formulation demonstrated good stability, caused no ocular irritation and promoted prolonged drug release for up to 18 h, along with precorneal retention lasting up to 40 min [96]. HPMC can also be used to enhance gel strength.

The findings of Jiang and co-workers [97] showed that berberine hydrochloride, a poorly water-soluble active compound, lowered the gelation temperature of P407 hydrogels and improved corneal adhesion, further reducing the gel erosion rate while modulating drug release [97]. The addition of HPMC enhanced gel strength due to its strong interaction with P407.

Paul et al. evaluated nine in situ gel formulations to identify the best option for enhancing the ocular delivery of ganciclovir. The optimized formulation, made up of 15% *w*/*v* P407 and 1% *w*/*v* HPMC E-50 LV, notably improved the eye permeation of the drug and prolonged its ocular retention time. Moreover, it enhanced the therapeutic efficacy of ganciclovir and it represents a promising alternative to conventional eye drops, offering a better patient compliance and effectiveness [98].

Chandler et al. [99] investigated a novel biodegradable, in situ thermo-responsive hydrogel proposed for the ocular surface healing. This formulation, made up of P407 (18.0 *w*/*v*%), P188 (5.0 *w*/*v*%) and HPMC (1.0 *w*/*v*%), was used to encapsulate human recombinant (rh)MG53 protein, a macromolecule already known for its capacity to promote corneal healing. The daily application of this system significantly improved the pharmacological activity of the protein and the related-healing phenomena [99].

In a promising new formulation proposed for wound healing, P407 was combined with alginate and foetal bovine serum to deliver the repurposed drug raloxifene. The system was investigated for its ability to influence cell proliferation, thereby facilitating rapid wound closure within 14 days. In-depth studies revealed that this formulation maximizes the proliferative potential of raloxifene. Further in vitro and in vivo analyses confirmed the efficacy and non-toxic nature of the formulation [100]. Histopathological studies revealed that P407 hydrogels promote collagen deposition, epithelial regeneration and neovascularization [101]. This evidence suggests that the poloxamer may act not only as a simple excipient, but as an active component characterized by wound healing features [102].

Poloxamers are generally valued in drug delivery formulations for antibiotics, particularly in the treatment of bacterial keratitis, an acute corneal infection caused by microorganisms such as Staphylococcus aureus. Conventional antibiotic formulations must be administered frequently to maintain effective antibacterial activity [20]. Suboptimal concentrations of these drugs may result in treatment failure or the emergence of bacterial resistance [103]. P407 is capable of inhibiting bacterial adhesion, including that of P. aeruginosa, through the formation of a hydrated layer on the surface of the microorganism [104].

In another in vivo study, an infected group of albino rabbits showed improved clinical outcomes after being treated with a specific bacteriophage, phage vB_Pa_ZCPS1, administered by an in situ gel. The formulation made up of 14% *w*/*v* P407 and 1.5% *w*/*v* HPMC K4M demonstrated a gelling time of 30 ± 1 s at a temperature of 33 °C ± 1 and useful muco-adhesive features [105].

A hybrid hydrogel was designed to effectively address ocular inflammation and infection through the localized co-delivery of antibiotics and corticosteroids. The system was obtained combining gellan gum (GG) (0.1% *w*/*v*) with various concentrations of P407 (16.5, 17 and 18% *w*/*v*). The formulation was enriched with polymyxin B sulphate, neomycin sulfate dexamethasone-loaded chitosan nanoparticles. The analyses revealed that GG positively influenced the system decreasing the gelation temperature and increasing the viscosity of P407 hydrogels. These effects contributed to obtain a sustained release of entrapped drugs enhanced their bioavailability, promoting potentially useful clinical outcomes [106].

Flurbiprofen is an anti-inflammatory drug also used to prevent miosis during eye surgery and to treat post-operative ocular inflammation. However, its poor water solubility presents a challenge [107]. To address this issue, nanoparticles composed of HPMC were fabricated using a spray-drying technique [108]. These nanoparticles were then dispersed in aqueous solutions containing 14% *w*/*w* P407 and Carbopol 934, leading to the development of innovative nanogels [109].

Owing to their gelling properties and extensive surface area, nanogels can interact with the ocular mucosa, facilitating enhanced administration of ophthalmic drugs [110,111]. In general, polymeric nanosystems can provide mucoadhesive properties that help prevent drug removal from the conjunctiva [112].

Fungal eye infections are another group of diseases that can affect ocular health. The antifungal activity of an in situ gel formulation composed of hyaluronic acid and P407, containing voriconazole-loaded cubosomes, was compared to that of a dispersion of the free drug by measuring fungal growth inhibition zones using a modified disk-diffusion susceptibility assay. The gel formulation proved to be 3.89 times more effective than the free drug and enhanced the permeation of the active compound [113]. The polysaccharide helped reduce the gelation temperature, surface friction, ocular dryness, and keratoconjunctivitis. These effects contributed to enhanced cubosome permeation, demonstrating significant mucoadhesive and anti-inflammatory properties [113].

Significant antifungal activity against Candida albicans was achieved using a thermo-sensitive gel composed of three polymers: P407, P188 and HPMC, containing itraconazole nanocrystals. The underlying rationale is that P407 (at a concentration of 20% *w*/*v*) forms a gel at room temperature, while P188, due to its lower hydrophobic/hydrophilic ratio, modulates the in situ gelation temperature of the system and HPMC enhances mucoadhesive strength [114,115]. The resulting formulation has a gelation temperature of 35 °C, adequate adhesion strength, an appropriate pH, high drug content and optimal viscosity [115].

### 4.3. Poloxamer 188 Hydrogels for the Treatment of Ocular Diseases

Poloxamer 188 (P188), also known by the trade name Pluronic^®^, F-68, is composed of PEO-PPO-PEO triblocks with average lengths of 80-30-80, respectively. The hydrophobic and hydrophilic segments give P188 its non-ionic nature and surface-active properties. With a Hydrophilic-Lipophilic Balance (HLB) of 29, P188 contains fewer hydrophobic PPO units compared to P407, which has an HLB of 22. Studies have shown that poloxamers exhibit a decrease in the Zeta potential of nanosystems as the molecular weight increases (i.e., from P188 to P407) [67]. Moreover, the CMC of P188 is slightly higher than that of P407 [116]. P188 in water can form three distinct phases:

1. at concentrations lower than the CMC and at temperatures below the CMT, the polymer exists in solution as single unimers above CMC and CMT individual molecules aggregate to form micellar structures;
2. as the temperature increases further, the micellar structures alter their microstructure, forming a denser network due to a reduction in the hydrophilicity of the PPO residues compared to the PEO chains. Additionally, P188 solutions become soft solids at high temperatures. Therefore, a reversible liquid-to-solid transition occurs, representing the shift from a viscoelastic liquid to a soft solid [117].

One of the most important properties of P188 is its neuroprotective characteristic and its ability to restore the functions of various types of injured cells with permeable membranes [118]. In fact, the evaluation of the interaction between P188 and lipid monolayers and liposomal bilayers showed that the hydrophobic PPO blocks penetrate the hydrocarbon region of the lipid membrane, while the hydrophilic PEO blocks remain in the aqueous compartments [119]. In addition, it has been demonstrated that P188 inhibits P-glycoprotein efflux pumps in ocular tissues, promoting the residence time of the active compound in the eye and enhancing its efficacy [120,121].

In this regard, P188 was proposed to create a micellar solution of ferulic acid with the aim of improving its solubility, stability and corneal permeability [122].

In addition to its significant antioxidant activity, which helps counteract free radicals and promote corneal regeneration, ferulic acid has been shown to have good antimicrobial activity against both Gram-positive and Gram-negative bacteria [123].

Micelles of P188 released approximately 70% of ferulic acid in 4 h, while micelle-nanogel formulations, composed of hyaluronan and ε-polylysine, exhibited a leakage of about 50% of the bioactive compound in 6 h, with a prolonged release lasting up to 48 h [122]. Despite the physico-chemical differences between P188 and P407, both polymers are suitable for the development of thermo-sensitive hydrogels for in situ ophthalmic administration as a consequence of their favourable safety profile and inherent optical transparency.

A recent experimental study described the development of two hydrogels containing orthosiphon, proposed for ophthalmic application [124].

In particular, the two systems were composed of 21% P407 and P188 in amounts ranging from 4% to 5%. Although the formulations were homogeneous and clear, they had a brown coloration, but they exhibited a phase transition from a solution to a gel at eye temperature. Furthermore, both formulations demonstrated an optimal transition temperature, ranging from 33 °C to 37 °C, a prolonged release of the active compound for up to 12 h and an optimal pH for the ocular compartment (between 7.27 and 7.46) [124].

Another strategy used to improve the solubility of hydrophobic drugs and promote their transcorneal permeation is encapsulating them within nanostructured lipid carriers (NLCs) [125,126]. However, the criticism of this approach is related to the low corneal retention time [127].

Wu et al. [128] integrated NLCs containing flurbiprofen (FB) within a thermo-sensitive nanohydrogel (FB-NLC-Gel) composed of P188 and P407. The formulation demonstrated significant ophthalmic anti-inflammatory effects. In fact, in vivo studies showed that the system increased the bioavailability of FB while maintaining good concentrations in the aqueous humor after topical instillation [128].

A 332-fold increase in flurbiprofen solubility was also achieved through the direct solubilization of the drug within an in situ gel system composed of P407 and P188, containing a flurbiprofen solid dispersion [129].

Prednisolone (PRD) is a corticosteroid used to treat ocular inflammatory diseases caused by infection, allergy, immune-mediated inflammation, irritation, injury, or trauma of the eye. It is insoluble in water; therefore, a microgel composed of lipid nanosystems containing PRD embedded in thermo-responsive hydrogels of P188 and P407 was prepared [130]. The viscosity of in situ formulations is essential for easy instillation into the eye and rapid conversion into a gel, ensuring sustained drug release and appropriate residence time of the entrapped molecules on the ocular surface. These features are crucial for the treatment of bacterial and fungal infections, as they help maintain the MIC for the required duration.

For example, ketoconazole (KTZ) was complexed with sulfobutylether-β-cyclodextrins and embedded within in situ hydrogels composed of P407 (20% *w*/*w*), P188 (5% *w*/*w*) and Carbopol 934 (0.2% *w*/*w*). The formulation increased the corneal residence time of the active compound, preserving its pharmacological efficacy [131].

**Figure 5:**
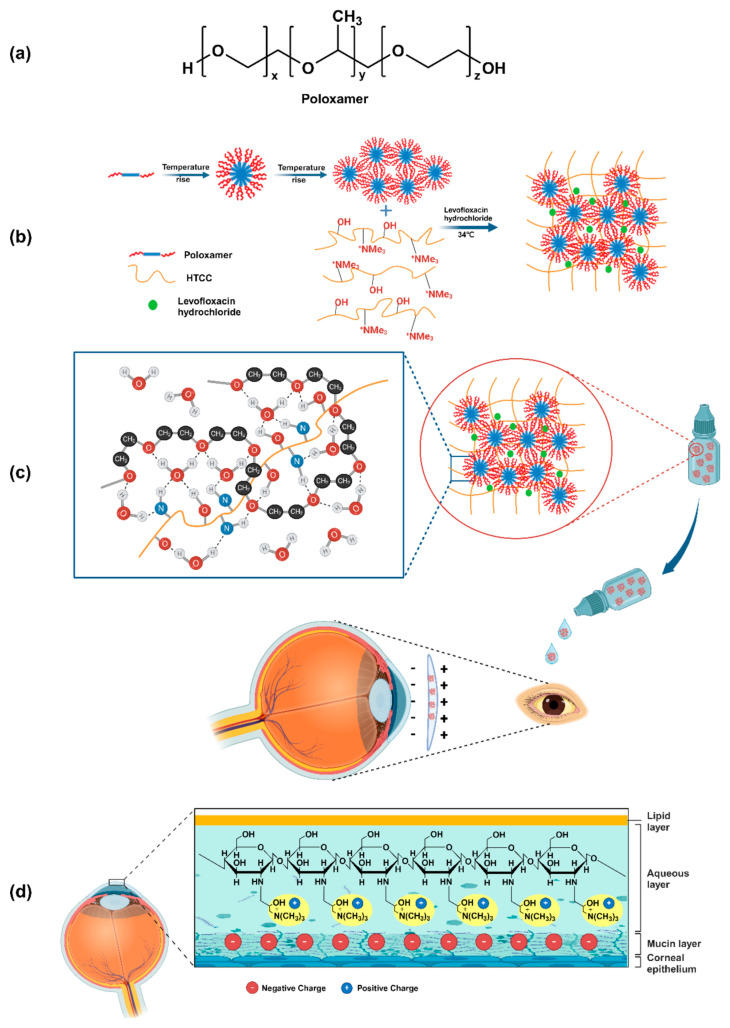
(**a**) Schematic representation of the poloxamer chemical structure, (**b**) mechanism of gelation for the in situ Poloxamer-HTCC system, (**c**) molecular structure of the in situ Poloxamer-HTCC gel and its utility in ocular drug delivery, (**d**) interaction with corneal mucins. Reproduced with permission from [132]. Copyright (2024) Elsevier.

P407 and P188 were used to synthesize a poloxamer-quaternized chitosan (Figure 5) utilizing N-(2-hydroxy-3-trimethylammonium) propyl chitosan chloride (HTCC) used to obtain a thermo-sensitive hydrogel able to promote a prolonged release of levofloxacin hydrochloride up to 48 h at 34 °C [132].

Recently, another thermo-sensitive multifunctional eye drop made up of P407, P188 and tannic acid, has shown promising results. The addition of tannic acid to the hydrogel can increase its adhesive ability to bind collagen and it significantly reduces the sol–gel transition temperature. This eye drop is capable of maintaining corneal retention for at least 90 min, a time significantly higher than 15 min of commercial muco-adhesive eye drops and 30 min of conventional in situ gels. The encapsulation of amphotericin B within the obtained system and the daily administration of the formulation showed a pharmacological efficacy six times higher than conventional drops of the drug [133].

A summary of all the previously discussed studies is provided in Table 2.

## 5. Characterization and Analysis of Thermosensitive In Situ Hydrogels for Ocular Applications

A key characteristic for ophthalmic formulations is clarity and the absence of particles, to prevent vision blurring after application. For this reason, the prepared in situ gel formulations are visually inspected for appearance and their clarity, colour uniformity, and particle presence are evaluated [134]. In addition to the aforementioned parameters, other characteristics must be carefully evaluated during the development of these formulations (Table 3).

Along with the appropriate selection of preservatives, ensuring the sterility of the final solution is essential for achieving a high-quality preparation. Sterilization methods include moist heat under pressure (autoclaving), dry heat, filtration, gas sterilization and ionizing radiation [135,136]. Sterilization by autoclaving in the final container (terminal sterilization) is feasible only for products whose stability remains unaffected [116,137].

Balu et al. [138] prepared a sterile thermo-sensitive gel of ofloxacin using P188 and HPMC, following the procedure outlined by the United States Pharmacopeia [139] (autoclaving at 121 °C, 15 psi, for 20 min). The evaluation of the physicochemical parameters before and after the procedure (flowability, pH, viscosity and sol–gel transition temperature) showed that no changes occurred. Autoclaving is the preferred method for sterilizing most ophthalmic solutions due to its efficiency and convenience in large-scale production. However, certain products cannot be sterilized using this method, as it may compromise their stability [116]. Therefore, if stability issues arise, an ophthalmic formulation can be sterilized by filtration through a 0.22 μm filter [140]. Even in this case, certain critical aspects must be considered, including the choice of filter, which must remain intact before and after use, the potential release of fibers and the degree of sample absorption on the filter [141].

According to the European Pharmacopoeia (Ph. Eur.) sterility test, a sample should be inoculated into a medium consisting of a mixture of soybean-casein digest broth (20 mL) and thioglycolate broth (20 mL). These media are then incubated for 14 days at 20 °C and 25 °C to assess potential microbial growth in both aerobic and anaerobic cultures [142].

Another important parameter to evaluate is tonicity. Osmolarity can be measured using a cryoscopic osmometer. Ideally, an ophthalmic formulation should have an osmolarity similar to that of natural tears (approximately 310 mOsm/kg), although the recommended upper limit is 340 mOsm/kg [143], with a tonicity equivalent to a 0.9% sodium chloride solution [144].

An essential parameter for ocular formulations, to prevent irritation and side effects, is pH, which should ideally range from 6.5 to 7.6. Although the eye can tolerate a pH range of 4.5 to 11.5 due to the buffering action of tear fluid, the optimal pH is 7.2 [145].

The irritation potential of an ophthalmic formulation is typically assessed using the Draize test. Specifically, 0.1 mL of the sample is instilled into the conjunctival cul-de-sac or applied directly to the cornea of an albino rabbit’s eye [30]. This test has been criticized for its poor reproducibility, the anatomical differences between human and rabbit eyes, and primarily for ethical reasons.

Consequently, one alternative used to replace the Draize test was the hen egg chorioallantoic membrane test (HET-CAM) [146]. The CAM is a highly vascularized extraembryonic structure containing arteries, capillaries and veins. This was considered an adequate model to predict the effects of substances on the conjunctiva of the eye, as adverse effects on the CAM induced by a test substance would correlate with irritation and/or corrosion in vivo [146].

In 2024, a research team developed a 3D device called OphthalMimic that incorporates artificial tear flow, a cul-de-sac area, a movable eyelid and a surface that effectively interacts with ophthalmic formulations. This provides a faithful representation of human ocular conditions, aiming to reduce animal testing [146]. Currently, however, an important application of such a device would be for dissolution/release tests that closely mimic in vivo conditions [147].

Two parameters of gelation warrant further investigation. One crucial parameter is the sol–gel transition temperature, which is frequently assessed using the tube inversion method. This procedure involves the gradual heating of the copolymer solution, typically in a vial or test tube, periodically inverting it. The gelation temperature is defined as the specific temperature at which the solution stops to flow upon inversion, marking its transition from a liquid to a solid-like (gel) state [72]. Ideally, for ocular applications, the sol–gel transition should occur at a temperature close to the ocular surface temperature, which in healthy individuals is usually 35.06 ± 1.24 °C [148]. In addition, the influence of lacrimal dilution on the gelation of the system should also be investigated in order to define ideal concentration of poloxamer to be employed for the development of the formulation [149]. Therefore, a dilution with simulated tear fluid is typically performed (added at 7:30 ratio corresponding to the ratio between the volume of physiological tear fluid and the average on an eye drop) to better simulate the in vivo conditions, assessing the gelation temperature [150].

The second parameter is the gelling capacity, which is determined by evaluating the time required for gel formation and the time required for its dissolution. Ideally, the formulation would exhibit an immediate sol–gel transition and subsequently dissolve over an extended period of hours. This characteristic would result in prolonged contact time and, consequently, a sustained drug effect [151].

The viscosity and rheological properties represent other fundamental aspects requiring evaluation for an in situ forming drug delivery system [152]. In particular, based on dynamic properties such as elasticity, storage modulus (G′) and loss modulus (G″), the characteristics of a formulation at both room and physiological temperatures must be thoroughly assessed. Under optimal conditions, the formulation should exhibit a free-flowing liquid state upon application, allowing for facile instillation as eye drops. Consequently, G″ should be greater than G′ at the time of application. Conversely, following administration, gel formation is anticipated; therefore, G′ should be greater than G″ to withstand the shear forces exerted by the eye during blinking [153].

The viscosity of the ophthalmic formulation is an important factor in the determination of residence time of drug in the eye and it can be determined by using a rheometer. The viscosity of an ophthalmic formulation is a critical parameter influencing the residence time of a drug within the eye and can be quantified using a rheometer [154].

Clearly, other established tests focusing on the evaluation of the release profile of active compounds within the formulations, their physical stability over a period of time, and their safety profile are all mandatory considerations during the preformulation stage of a thermo-sensitive hydrogel intended for ocular application [155,156].

**Table 3:** Main parameters to be investigated for the characterization of thermo-sensitive hydrogels for ocular applications.

Parameter to Be Evaluated	Objective and Method of Analysis	Ideal Key Outcome	References
Appearance and Clarity	Visual inspection for clarity, color uniformity and absence of particles to prevent vision blurring.	Clear, uniform formulation with no particles.	[134]
Sterility	Evaluation of the absence of microbial contamination. Sterilization methods include autoclaving and filtration. The European Pharmacopoeia test involves incubating the sample for 14 days in specific media.	The formulation remains sterile with no physical or chemical alterations.	[138,139,142]
Osmolarity	Measurement of osmolarity using a cryoscopic osmometer. The goal is to prevent irritation.	Osmolarity similar to that of natural tears (approximately 310 mOsm/kg), with a recommended upper limit of 340 mOsm/kg.	[143,144]
pH	pH measurement to prevent irritation and side effects.	Ideal pH range between 6.5 and 7.6, with an optimal pH of 7.2.	[145]
Sol–Gel Transition	Evaluation of the temperature at which the transition from liquid to gel occurs, often using the tube inversion method.	Transition at ocular surface temperature after instillation.	[72,148]
Gelling Capacity	Evaluation of the time required for gel formation and dissolution.	Rapid gel formation and prolonged dissolution over several hours, for extended contact time.	[151]
Viscosity and Rheology	Measurement of viscosity and rheological properties (G′ and G″ moduli) with a rheometer to determine drug residence time.	The formulation should be a free-flowing liquid (G″ > G′) at room temperature for easy application and transform into a gel (G′ > G″) at physiological temperature to withstand blinking forces.	[152]
Physical Stability and Drug Release	Evaluation of the release profile of the active compound(s) and physical stability over time.	Prolonged drug release and long-term stability of the formulation.	[155,156]
Ocular Irritation	Evaluation of irritation potential. The Draize test on albino rabbits is the traditional method.	Low or no irritation potential.	[30]

## 6. Conclusions

Ocular diseases present a significant therapeutic challenge due to the diverse anatomical biobarriers inherent to this bodily compartment. The rising global prevalence of these conditions, coupled with the severe risk of vision impairment or blindness in the absence of treatment, underscores the pressing need for innovative therapeutic strategies [157]. Furthermore, the prevalence of eye diseases imposes a substantial burden on healthcare systems; for instance, dry eye disease (DED) is estimated to incur costs of nearly $4 billion annually within the US healthcare system [158].

Academic research is fundamental for developing new therapeutic approaches and innovative formulations. However, translating proposed systems to a scale-up process can be challenging, as was the case with nanoparticles that often required complex and expensive manufacturing and specific equipment [159,160].

One advantage of poloxamer-based hydrogels is their quick and easy preparation using the cold method [161]. Ideally, the constituents of a hydrogel should be available in GMP quality at reasonable costs and possess properties such as biocompatibility, biodegradability and approval for human use [160]. Despite numerous experimental investigations, only a limited number of ophthalmic formulations incorporating P407 have obtained clinical approval from the FDA and EMA, as is the case with AzaSite Plus^®^ (from Inspire Pharmaceuticals) and BromSite^®^ (from Sun Pharmaceutical Industries) [85].

It is essential to emphasize that P407 is combined with other polymers, such as Polycarbophil, within these formulations to augment its inherent properties [24]. This synergy is intended to optimize the in situ gelation and mucoadhesive properties of the system, which are essential characteristics for the effective treatment of ocular inflammation and infections through the promotion of prolonged drug contact with the ocular surface [157].

Considering the aforementioned aspects and the experimental findings detailed in this document, thermo-responsive hydrogels composed of P407 and P188 present viable drug delivery systems for the treatment of conditions affecting the anterior segment of the eye.

## Figures and Tables

**Table 1:** Parameters used for the classification of hydrogels.

Source	Composition	Crosslinking	Configuration	Ionic Charge	Property	Response
Natural	Homopolymer	Physical	Amorphous	Non-ionic	Conventional	Physical stimuli
Synthetic	Copolymer	Chemical	Crystalline	Anionic	Intelligent	Chemical stimuli
Semi-synthetic	Semi-IPN		Semi-crystalline	Cationic		
	IPN			Ampholytic		

Abbreviations: IPN, interpenetrating polymer networks.

**Table 2:** Main characteristics of poloxamer-based in situ hydrogels for treating ocular diseases.

Type and Amount of Poloxamer Used	Other Components	Type of Hydrogel	Type of Embedded Carrier	Active Compound	Applications	References
P407 15% *w*/*w*	HPMC 1.5% *w*/*w*	E	Niosomes	Doxycycline	Antibacterial activity	[72]
P407 8.16% *w*/*v*	HPMC 0.77% *w*/*v*	G	-	Chloramphenicol	Antimicrobial activity	[95]
P407	HPMC	G	-	Berberine	Antitumor activity; Antibiotic property; Antioxidant; Anti-inflammatory effects; Gastroenteric discomfort; Diabetes in clinic	[97]
P407 17.5–22.5% *w*/*v*	Chitosan	G	-	Tacrolimus	Allograft corneal rejection; Mooren’s ulcer; Allergic conjunctivitis; Immunogenic inflammatory ocular surface diseases; Posterior uveitis; Posterior blepharitis	[93]
P407 P188	HPMC K4M	G	-	Nifedipine	Glaucoma	[40]
P407 15–20% *w*/*v* P188 0–7.50% *w*/*v*	HPMC 0.5–1.5% *w*/*v*	E	Nanocrystals	Itraconazole	Fungal infections	[115]
P407 14% *w*/*w*	Carbopol 934 0.3% *w*/*w*	E, N	HPMC nanoparticles	Flurbiprofen	Prevention of miosis throughout ocular surgery; Postoperative ocular inflammation	[109]
P7: P407 21% *w*/*v* P188 4% *w*/*v*; P8: P407 21% *w*/*v* P188 5% *w*/*v*	F5: chitosan 1.5% *w*/*v* β-glycerophosphate 45% *w*/*v*	G	-	Orthosiphon Stamineus Benth	Antimicrobial activity	[124]
F2: P407 15% *w*/*w* HA 0.2% *w*/*w* F4: P407 10% *w*/*w* HA 0.4% *w*/*w*	-	E	Cubosomes	Voriconazole	Fungal infections	[113]
P407 27.36% *w*/*v* P188 6.22% *w*/*v*	-	E, N	Nanostructured lipid carriers	Flurbiprofen	Anti-inflammatory therapy	[128]
P407 10% and 12% *w*/*w* P407 12% *w*/*w* and P188 1–10% *w*/*w*	-	E, M	Lipid nanosystems oil-in-water (O/W) microemulsion	Prednisolone	Ocular inflammatory disease	[130]
P407 20% *w*/*w* P188 5% *w*/*w*	Carbopol 0.2% *w*/*w*	G		Ketoconazole complexed with sulfobutylether-β-cyclodextrin	Fungal infections	[131]
P407 20% wt	-	G	-	Progranulin	Anti-inflammatory action; Regeneration and re-epithelialization of corneal tissue	[66]
P407 20.5% *w*/*v* P188 5.0% *w*/*v*	-	E	Chitosan nanoparticles	Chloramphenicol	Antimicrobial activity	[96]
Aldehyde-functionalized P407 (AF127 different concentrations)	-	G	Polyvinylpyrrolidone nanoparticles	$Cu_{2-x}$Se	Antioxidant; Dry eye disease	[90]
P407 P188	N-(2-hydroxy-3-trimethylammonium) propyl chitosan chloride	M	-	Levofloxacin hydrochloride	Fungal keratitis	[132]
P407	Alginate	E, N	Carboxymethyl chitosan nanosuspension	Clobetasol propionate	Uveitis treatment	[86]
P407-based gel (SEP)	Selenol	M	Nanosuspension	Fluticasone propionate	Blepharitis	[89]
P407 15% *w*/*v*	HPMC E-50 LV 1% *w*/*v*	G	-	Ganciclovir	Cytomegalovirus retinitis; Herpetic keratitis	[98]
P407 18.0 *w*/*v*% P188 5.0 *w*/*v*%	HPMC (1.0 *w*/*v*%)	G	-	(rh)MG53 protein	Promote corneal healing	[99]
P407 14%	HPMC K4M 1.5%	E	Bacteriophage	phage vB_Pa_ZCPS1	Pseudomonas aeruginosa keratitis	[105]
P407 16.5, 17, and 18% *w*/*v*	Gellan Gum 0.1% *w*/*v*	E, M	Chitosan nanoparticles	Dexamethasone Polymyxin B sulfate Neomycin sulfate	Inflammation and infection	[106]
P407 P188	Tannic Acid	G	-	Amphotericin B	Corneal ulcers	[133]
P407 10–20% *w*/*v*	Alginate	G	-	Raloxifene	Wound healing	[100]

Abbreviations: P407, poloxamer 407; P188, poloxamer 188; HPMC, hydroxypropyl methyl cellulose; HA, hyaluronic acid; E, embedded: thermo-responsive hydrogel containing nanosystems; G, thermo-responsive hydrogel; M, microhydrogel; N, nanohydrogel.

## Data Availability

No new data were created or analyzed in this study.
